# *In Vivo* Evidence of Single ^13^C and ^15^N Isotope–Labeled Methanotrophic Nitrogen-Fixing Bacterial Cells in Rice Roots

**DOI:** 10.1128/mbio.01255-22

**Published:** 2022-05-24

**Authors:** Shintaro Hara, Naohisa Wada, Sliver Sung-Yun Hsiao, Meng Zhang, Zhihua Bao, Yoshiyuki Iizuka, Der-Chuen Lee, Shusei Sato, Sen-Lin Tang, Kiwamu Minamisawa

**Affiliations:** a Graduate School of Life Sciences, Tohoku University, Sendai, Japan; b Institute for Agro-Environmental Science, National Agriculture and Food Research Organization (NARO), Tsukuba, Japan; c Biodiversity Research Center, Academia Sinica, Taipei, Taiwan; d Institute of Astronomy and Astrophysics, Academia Sinica, Taipei, Taiwan; e Inner Mongolia Key Laboratory of Environmental Pollution Prevention and Resource Recycle, Hohhot, China; f Institute of Earth Science, Academia Sinica, Taipei, Taiwan; CEH-Oxford

**Keywords:** diazotrophy, methanotrophy, NanoSIMS, paddy rice, single cell, type II methanotrophs

## Abstract

Methane-oxidizing bacteria (methanotrophs) play an ecological role in methane and nitrogen fluxes because they are capable of nitrogen fixation and methane oxidation, as indicated by genomic and cultivation-dependent studies. However, the chemical relationships between methanotrophy and diazotrophy and aerobic and anaerobic reactions, respectively, in methanotrophs remain unclear. No study has demonstrated the cooccurrence of both bioactivities in a single methanotroph bacterium in its natural environment. Here, we demonstrate that both bioactivities in type II methanotrophs occur at the single-cell level in the root tissues of paddy rice (Oryza sativa L. cv. Nipponbare). We first verified that difluoromethane, an inhibitor of methane monooxygenase, affected methane oxidation in rice roots. The results indicated that methane assimilation in the roots mostly occurred due to oxygen-dependent processes. Moreover, the results indicated that methane oxidation-dependent and methane oxidation-independent nitrogen fixation concurrently occurred in bulk root tissues. Subsequently, we performed fluorescence *in situ* hybridization and NanoSIMS analyses, which revealed that single cells of type II methanotrophs (involving six amplicon sequence variants) in paddy rice roots simultaneously and logarithmically fixed stable isotope gases ^15^N_2_ and ^13^CH_4_ during incubation periods of 0, 23, and 42 h, providing *in vivo* functional evidence of nitrogen fixation in methanotrophic cells. Furthermore, ^15^N enrichment in type II methanotrophs at 42 h varied among cells with an increase in ^13^C accumulation, suggesting that either the release of fixed nitrogen into root systems or methanotroph metabolic specialization is dependent on different microenvironmental niches in the root.

## OBSERVATION

Methane is a powerful greenhouse gas, and atmospheric methane concentrations are increasing rapidly (1). However, methane-oxidizing bacteria (methanotrophs) can reduce methane fluxes and thus mitigate climate change (1). Methanotrophs are divided mainly into two major phylogenetic groups: type I (*Gammaproteobacteria*) and type II (*Alphaproteobacteria*) (2, 3). Many methanotrophs are involved in nitrogen fixation (3–5) and thus may participate in environmental nitrogen cycling (3). Indeed, the nitrogenase structural genes *nifHDK* were completely encoded on 88.8% of the genomes of type I methanotrophs and on 98.3% of the genomes of type II methanotrophs in the current publicly available methanotrophic genomes (80 and 37 genomes, respectively; see Table S1 in the supplemental material), suggesting that most of methanotrophs, especially type II, could drive nitrogen fixation. Furthermore, the nitrogenase gene (*nifH*) sequences of both types of methanotrophs have been detected from terrestrial, freshwater, and marine environments across the world (Fig. S1), suggesting that diazotrophic methanotrophs may be ubiquitously distributed worldwide.

10.1128/mbio.01255-22.1 FIG S1 Geographic and habitat distributions of methanotrophs based on the *nifH* gene in the families *Methylococcaceae* (type I methanotrophs) and *Methylocystaceae* (type II methanotrophs). The *nifH* genes, encoding nitrogenase reductase, were retrieved from the NCBI database and carefully examined as *nifH* genes of *Methylococcaceae* and *Methylocystaceae*. The map (A) and habitat table (B) illustrate the features noted in the data. *Methylocystaceae* (type II methanotrophs) carrying the *nifH* gene were widely distributed in many countries (green and yellow in A) and in terrestrial environments, including freshwater habitats (gray in B). Download FIG S1, PDF file, 0.6 MB. Copyright © 2022 Hara et al. 2022 Hara et al. https://creativecommons.org/licenses/by/4.0/ This content is distributed under the terms of the Creative Commons Attribution 4.0 International license.

10.1128/mbio.01255-22.6 TABLE S1 Presence/absence of methane monooxygenase and nitrogen fixation structural genes (*nifHDK*) in available genomes of methanotrophs. Download Table S1, PDF file, 0.2 MB. Copyright © 2022 Hara et al. 2022 Hara et al. https://creativecommons.org/licenses/by/4.0/ This content is distributed under the terms of the Creative Commons Attribution 4.0 International license.

Rice paddy fields are a hot spot for methane metabolism and a habitat of type II methanotrophs (6–8). Methane monooxygenase (MMO) and nitrogenase of type II methanotrophs were simultaneously expressed in rice root-associated bacteria in a low-N paddy field (6). Type II methanotrophs exhibit an endophytic lifestyle in the vascular cylinders and epidermal cell layers of root tissues (6, 9). Nitrogenase for nitrogen fixation requires anoxic conditions, whereas bacterial methane oxidation in type II methanotrophs requires molecular oxygen. Pure culture experiments demonstrated that type II methanotrophs isolated from rice roots fix nitrogen in a methane-dependent manner (methane oxidation-dependent nitrogen fixation) (10). However, oxygen is a key element in both of these reactions. Oxygen regimens in rice roots in paddy fields vary from the aerobic vascular cylinder to the anoxic epidermis (11). This leads to the creation of heterogenous microenvironments with different oxygen levels that affect methane oxidation-dependent nitrogen fixation by individual microbial cells in these microenvironmental niches. Although studies have identified the specific metabolic activities of methanotrophs, they used only test tube-based homogenous methanotroph cultures (4, 10). Therefore, whether methanotrophs can mediate methane oxidation and nitrogen fixation simultaneously at the single-cell level in their natural environments remains unclear (12).

Formaldehyde (HCHO) is a central intermediate in methanotroph metabolism, and its carbon sources are derived from methane through a dissimilation pathway (CH_4_→CH_3_OH→HCHO) mediated by (i) methane monooxygenase and methanol dehydrogenase and (ii) subsequent HCHO assimilation, such as that in the serine pathway (2). This pathway indicates that ^13^CH_4_ can enrich methanotroph compounds in ^13^C.

It would be difficult to conduct an *in situ* experiment in a paddy field due to the continuous production of methane from organic matter by methanogens living in anaerobic soil and the rice rhizosphere, and this would dilute the ^13^C concentration of the methane by simple isotope addition. Although slightly different from the natural condition, in this study, we performed *in vivo* stable isotope labeling experiments by using paddy rice roots and single-cell imaging (fluorescence *in situ* hybridization [FISH] and NanoSIMS). Our results provide *in vivo* functional evidence of nitrogen fixation by type II methanotrophs residing in the root tissues of paddy rice at the single-cell level (details of root preparation, stable isotope feeding, mass spectrometry, bacterial cell extraction, amplicon sequencing, and FISH-NanoSIMS analyses are provided in Text S1).

10.1128/mbio.01255-22.10 TEXT S1 Supplementary materials and methods. Download Text S1, PDF file, 0.3 MB. Copyright © 2022 Hara et al. 2022 Hara et al. https://creativecommons.org/licenses/by/4.0/ This content is distributed under the terms of the Creative Commons Attribution 4.0 International license.

**FIG 1:**
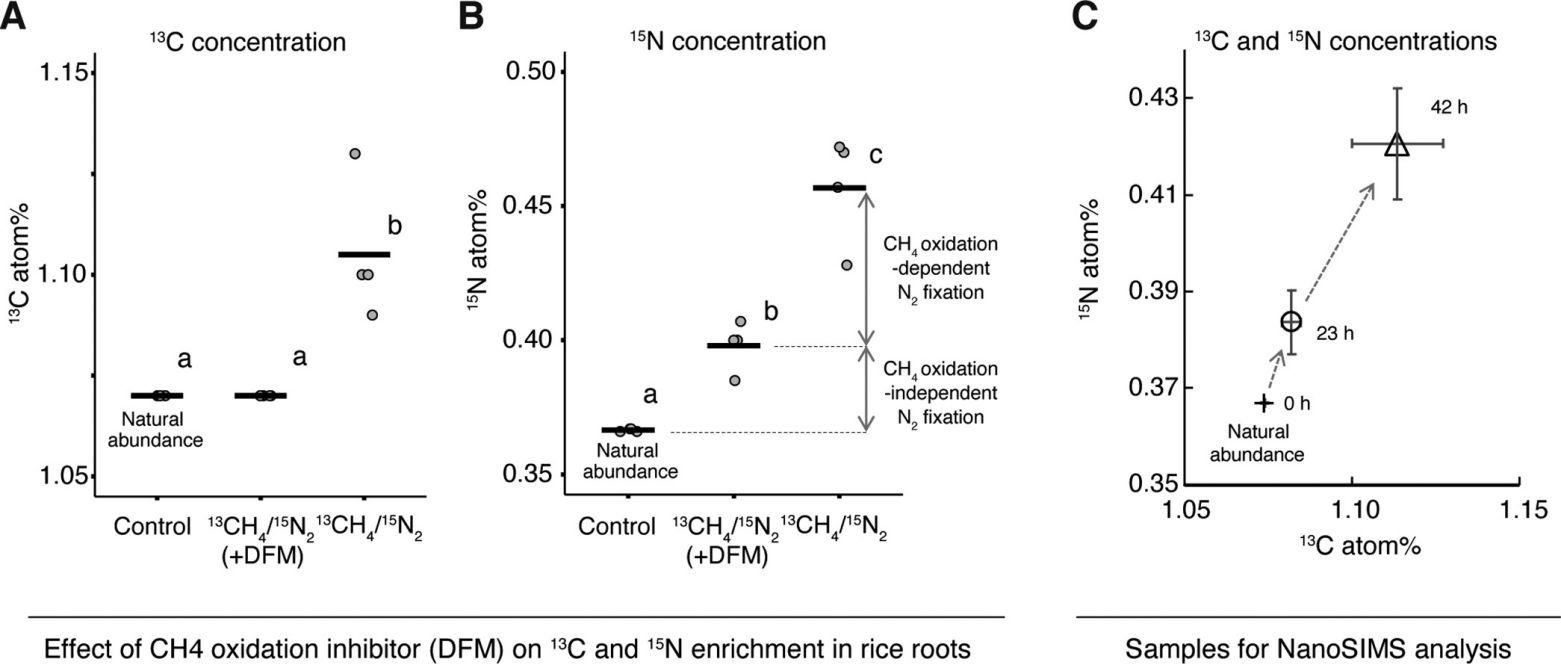
(A to C) ^13^CH_4_ and ^15^N_2_ concentrations in rice roots with and without methane oxidization inhibitor and the NanoSIMS experiment. (A and B) ^13^C (A) and ^15^N (B) concentrations of rice roots fed with ^13^CH_4_ (5% [vol/vol], 99 atom% ^13^C), ^15^N_2_ (39% [vol/vol], 40.8 atom% ^15^N), and 5% (vol/vol) O_2_ in Ar balance for 24 h with the addition of difluoromethane (DFM; 0.5% [vol/vol]), a methane monooxygenase inhibitor; “control” indicates root samples before isotope feeding. Average values with the same letter are not significantly different according to Tukey’s honestly significant difference test (*P < *0.05). (C) ^13^C and ^15^N concentrations in the root samples determined by performing NanoSIMS analysis, with the root systems of field-grown rice plants incubated with a gas phase containing ^13^CH_4_ (6% [vol/vol], 99 atom% ^13^C), ^15^N_2_ (35% [vol/vol], 99.4 atom% ^15^N), and O_2_ (12% [vol/vol]) in Ar balance for 0, 23, and 42 h. Bolded horizontal bars in A and B indicate the averages of four replicates.

The root systems of rice plants grown in a paddy field were incubated with ^13^CH_4_/^15^N_2_ for 24 h with and without difluoromethane (DFM), a methane monooxygenase inhibitor (13). A low concentration of DFM is known to effectively and selectively inhibit methanotrophy by competing as a substrate for MMO (13, 14). ^13^C concentrations in roots exposed to ^13^CH_4_/^15^N_2_ with DFM were identical to the natural abundance of ^13^C (control, 1.07 atom%) in rice roots, and ^13^C concentrations in the samples without DFM were significantly higher than the natural abundance level. The enrichment of ^13^C in the sample without DFM could indicate oxygen-dependent ^13^CH_4_ oxidation and assimilation by methanotrophs residing in the roots (Fig. 1A). ^15^N concentrations in roots exposed to ^13^CH_4_/^15^N_2_ with DFM were significantly higher than the natural abundance of ^15^N (control, 0.366 atom%). Moreover, the absence of DFM significantly increased ^15^N concentrations in the roots exposed to ^13^CH_4_/^15^N_2_ (Fig. 1B). These results suggest that both methane-dependent and methane-independent nitrogen fixation occur in paddy rice roots. On the basis of differences in ^15^N concentrations in the rice roots with and without DFM, methane-dependent nitrogen fixation was determined to be 0.49 μmol N_2_ fixed (g root weight)^−1 ^day^−1^, accounting for 65% of total nitrogen fixation (Table S2).

10.1128/mbio.01255-22.7 TABLE S2 Nitrogen fixation in rice roots estimated by ^15^N enrichment and N content. Download Table S2, PDF file, 0.2 MB. Copyright © 2022 Hara et al. 2022 Hara et al. https://creativecommons.org/licenses/by/4.0/ This content is distributed under the terms of the Creative Commons Attribution 4.0 International license.

For the NanoSIMS analysis, field-grown rice roots were incubated again with a gas containing ^13^CH_4_/^15^N_2_ (99.4 atom% ^15^N) for 0, 23, and 42 h. The concentrations of both ^13^C and ^15^N increased with incubation time, suggesting that methanotrophic nitrogen fixation occurred in the root samples (Fig. 1C). We then subjected bacterial cells extracted from the rice root tissues (15) to FISH-NanoSIMS analyses. The amplicon sequences of the 16S rRNA gene of the bacterial cells indicated an abundance of type II methanotrophs (*Methylocystaceae*; average, 7.2%), including six amplicon sequence variants (ASVs; Table S3A and B) that were phylogenetically split equally into two genera (*Methylocystis* and *Methylosinus*) (Fig. S2); ASV0004 (belonging to *Methylosinus*) was most dominant among the samples (average, 6.61%; Table S3A and Fig. S2). In contrast, type I methanotrophs were assigned with only a single ASV (belonging to *Methylococcus*; average abundance, 0.1%) (Table S3A and Fig. S3). All methanotrophic ASVs were widely positioned in the phylogenetic tree, and each ASV was close to a respective genome that presented particulate MMO (pMMO), soluble MMO (sMMO), and the *nifHDK* gene cluster (Fig. S2 and S3), suggesting that all ASVs could potentially participate in both methane oxidation and nitrogen fixation. We also confirmed that all ASV sequences of type II methanotrophs were identical to the sequence of the FISH probe Ma450 from a previous study (16), and no other bacterial ASVs were not matched to the probe Ma450. The FISH analyses performed using the Ma450 probe for type II methanotrophs (16) and the EUB338mix probe for total eubacteria (17) indicated that the proportion of type II methanotrophs in total bacterial cells ranged from 6.1% to 7.2% (Fig. S4); this result is in agreement with the amplicon sequences of the 16S rRNA gene.

10.1128/mbio.01255-22.2 FIG S2 Phylogenetic analysis of the 16S rRNA gene from six amplicon sequence variants (ASVs) and available genomes of type II methanotrophs. Bootstrap values greater than 50% derived from 1,000 replicates are also shown as black dots. Colored symbols indicate which sequences are matched to a Ma450 probe (green) and which genomes contained particulate methane monooxygenase (pMMO; blue), soluble methane monooxygenase (sMMO; light blue), and the three nitrogenases *nifH* (yellow green), *nifD* (orange), and *nifK* (red). Download FIG S2, PDF file, 0.2 MB. Copyright © 2022 Hara et al. 2022 Hara et al. https://creativecommons.org/licenses/by/4.0/ This content is distributed under the terms of the Creative Commons Attribution 4.0 International license.

10.1128/mbio.01255-22.3 FIG S3 Phylogenetic analysis of the 16S rRNA gene from one amplicon sequence variants (ASV) and available genomes of type I methanotrophs. Bootstrap values greater than 50% derived from 1,000 replicates are also shown as black dots. Colored symbols indicate which genomes contained particulate methane monooxygenase (pMMO; blue), soluble methane monooxygenase (sMMO; light blue), and the three nitrogenases *nifH* (yellow green), *nifD* (orange), and *nifK* (red). Download FIG S3, PDF file, 0.2 MB. Copyright © 2022 Hara et al. 2022 Hara et al. https://creativecommons.org/licenses/by/4.0/ This content is distributed under the terms of the Creative Commons Attribution 4.0 International license.

10.1128/mbio.01255-22.4 FIG S4 Proportion (%) of cells identified as type II methanotrophs (green) and other eubacteria (red) based on FISH images at all time points (0, 23, and 42 h). Download FIG S4, PDF file, 0.1 MB. Copyright © 2022 Hara et al. 2022 Hara et al. https://creativecommons.org/licenses/by/4.0/ This content is distributed under the terms of the Creative Commons Attribution 4.0 International license.

10.1128/mbio.01255-22.8 TABLE S3 Relative abundance of type I and II methanotrophs (A) and major abundance sequence variants (ASVs) (B) extracted from rice roots (at 0 h). Table S3, XLSX file, 0.03 MB Copyright © 2022 Hara et al. 2022 Hara et al. https://creativecommons.org/licenses/by/4.0/ This content is distributed under the terms of the Creative Commons Attribution 4.0 International license.

**FIG 2:**
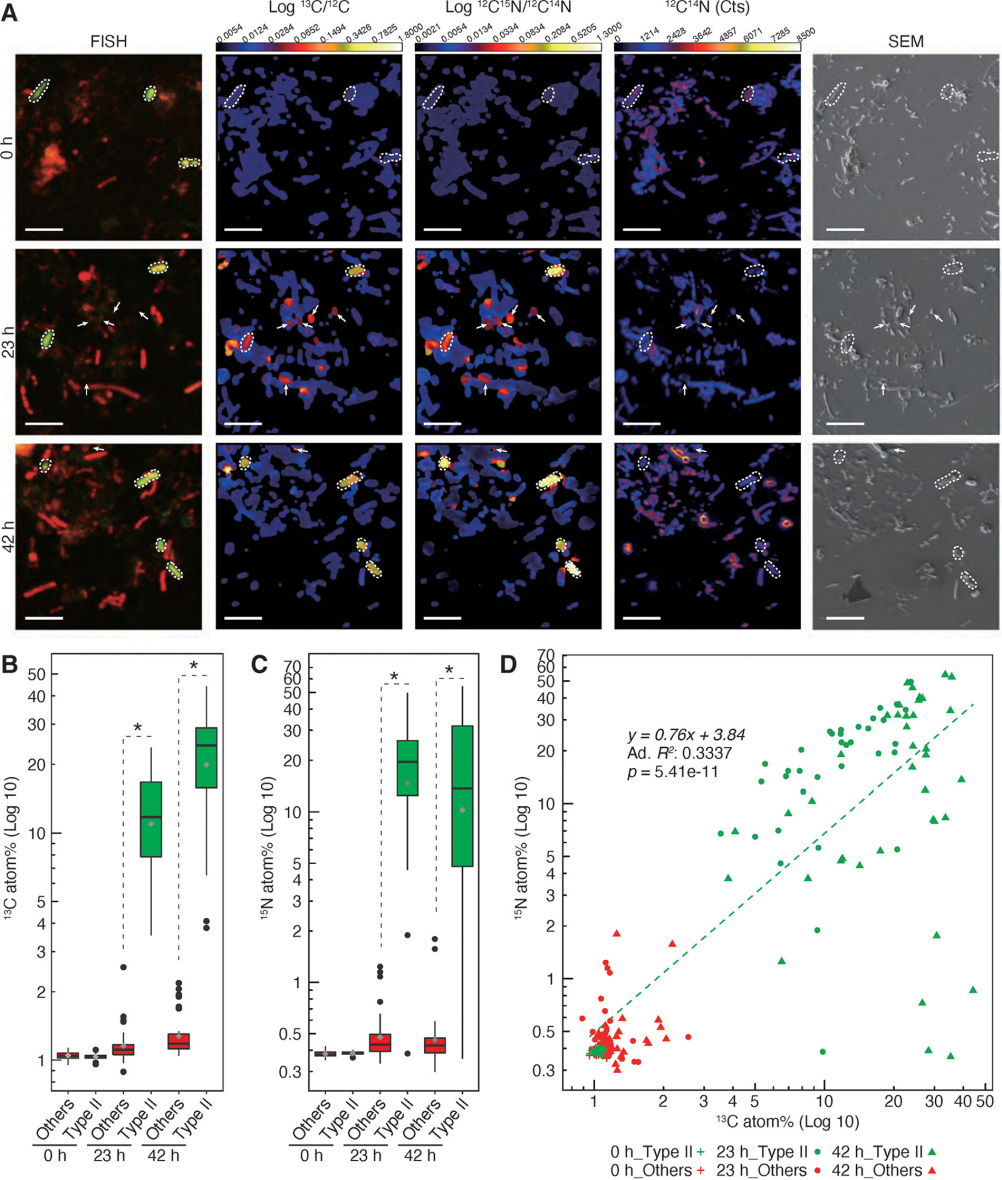
Methane assimilation and nitrogen fixation of type II methanotrophs and other eubacteria in rice roots at the single-cell level. (A) Example parallel images of FISH, carbon isotope ratio (log_10_ [^13^C/^12^C]), nitrogen isotope ratio (log_10_ [^12^C^15^N/^12^C^14^N]), ^12^C^14^N counts, and scanning electron micrographs (SEM) for symbiotic microbes in rice roots at 0 h, 23 h, and 42 h of incubation. Green fluorescence in FISH images indicates type II methanotrophic bacteria, and red fluorescence indicates other eubacteria (hybridized with Ma450 and EUB338 mix probes labeled with Alexa 488 and Cy3, respectively). Type II methanotrophic cells hybridized with both probes (yellow signals indicated with white dashed lines). Arrows indicate regions with high ratios of carbon and nitrogen isotopes without FISH signals (suggesting dead cells as the cause because FISH targets rRNA in cells). Scale bars indicate 5 μm. (B and C) Statistics for carbon (B) (log_10_ [^13^C atom%]) and nitrogen (C) isotopic composition (log_10_ [^15^N atom%]) for type II methanotrophic bacteria and other eubacteria individuals. Asterisks indicate significant differences between type II methanotrophic bacteria and other eubacteria in unpaired two-sample Student’s *t* test (*P < *0.01). (D) Carbon (log_10_ [^13^C atom%]) and nitrogen isotopic composition (log_10_ [^15^N atom%]) for type II methanotrophic bacteria and other eubacteria individuals presented as a scatterplot. The linear regression indicates a significant positive correlation between ^13^C and ^15^N enrichment for type II methanotrophic bacteria at all time points (*P < *0.01). Ad. *R^2^* indicates Adjusted R-Squared.

The subsequent NanoSIMS analysis revealed the overlapping images of δ^13^C and δ^15^N signals in the cells that were merged using Ma450 probe signals in the 23-h and 42-h specimens (Fig. 2A). We determined ^13^C and ^15^N atom% of more than 100 cells in the NanoSIMS images, and a significant difference was observed in ^13^C and ^15^N concentrations between type II methanotrophs and other eubacteria. After stable isotope feedings, all type II methanotrophs exclusively enriched ^13^C and ^15^N concentrations, while other eubacteria did not (Fig. 2B and C). As depicted in the scatterplot in Fig. 2D, a significant positive correlation was noted between ^13^C and ^15^N enrichment for type II methanotrophs at all time points. For every mole of ^13^C that was assimilated, an average 0.76 mol of ^15^N were fixed at the single-cell level (Fig. 2D), which was three times higher than the previously reported value of 0.25 mol (18, 19).

Concentrations of ^13^C and ^15^N increased with high variability on a logarithmic scale at 23 and 42 h. Notably, a large fluctuation in the ^15^N enrichment of type II methanotrophs occurred among the cells at 42 h, and a slight saturation of ^13^C was noted (Fig. 2B and C). This finding indicates that at 42 h, individual type II methanotrophic cells enabled the accumulation of either ^13^C alone or both ^13^C and ^15^N in the root tissues (Fig. S5), which may be allowed by the creation of heterogeneous microenvironmental niches of type II methanotrophs, including in the vascular cylinders and epidermal cell layers of root tissues (6, 9). In addition, inter- or intraspecific variation of oxygen sensitivity in methanotrophs, mainly type II, has been reported (Table S4). It is also possible that different methanotrophic species or strains in the root system could differ in their sensitivity to oxygen. Given that the metabolic specialization of heterogeneous nitrogen fixation can occur at the single-cell level in diazotroph cyanobacteria (20), some type II methanotrophic cells also may transform into low- or nonnitrogen-fixing mode to save energy for creating an anoxic microenvironment.

10.1128/mbio.01255-22.5 FIG S5 Representative correlated FISH and NanoSIMS images indicating variations in ^15^N accumulation within type II methanotrophic cells (dotted lines) in the root tissue at 42 h after isotope incubation and ^13^C enrichment in both cells. (A) FISH panel of type II methanotrophs and other eubacteria hybridized with Ma450 (green, Alexa 488) and EUB338 mix (red, Cy3) probes. (B to E) NanoSIMS mapping images of carbon isotope ratio (^13^C/^12^C), nitrogen isotope ratio (^12^C^15^N/^12^C^14^N), ^12^C^14^N counts, and secondary electron (SE) micrographs. Scale bars represent 5 μm. Download FIG S5, JPG file, 2.4 MB. Copyright © 2022 Hara et al. 2022 Hara et al. https://creativecommons.org/licenses/by/4.0/ This content is distributed under the terms of the Creative Commons Attribution 4.0 International license.

10.1128/mbio.01255-22.9 TABLE S4 Variation of oxygen sensitivity in type I and II methanotrophs. Download Table S4, XLSX file, 0.01 MB. Copyright © 2022 Hara et al. 2022 Hara et al. https://creativecommons.org/licenses/by/4.0/ This content is distributed under the terms of the Creative Commons Attribution 4.0 International license.

Interestingly, this varied pattern of ^15^N enrichment in single cells differed from a marked increase in ^15^N and ^13^C concentrations observed up to 42 h in bulk root tissues (Fig. 1C). This outcome suggests the potential influence of several potential factors, such as the accumulation from other nitrogen fixers including type I methanotrophs and/or a release of fixed nitrogen (ammonium or organic nitrogen) from type II methanotrophs at the single-cell level into the root system. In fact, peatland methanotrophs can provide not only carbon but also nitrogen to peat mosses, suggesting carbon and nitrogen accumulation in the field (12). Although further work is need, our findings expand our knowledge of the intact carbon and nitrogen cycle at the single-bacterial-cell level, particularly in the paddy rice root system.

Because type II methanotrophs in intact root tissues accumulated stable isotopes from both ^13^CH_4_ and ^15^N_2_ gases at the single-cell level (Fig. 2D), root-associated type II methanotrophs might have simultaneously performed methane oxidation and assimilation and methane-dependent nitrogen fixation *in vivo* in the root tissues of paddy rice. Given that nitrogen fixation heterogeneously varied at the single-cell level, we hypothesize that type II methanotrophic cells contribute nitrogen flux to root systems after nitrogen fixation or affect root systems’ nitrogen accumulation through the creation of microenvironmental niches. Our findings provide insights into potential *in situ* interactions that occur between methanotrophy and diazotrophy in terrestrial carbon and nitrogen cycles (12, 21) as well as in agricultural settings (9, 18, 22).
