# Supplementary material for: In Vivo Evidence of Single 13C and 15N Isotope–Labeled Methanotrophic Nitrogen-Fixing Bacterial Cells in Rice Roots
Source: mBio. 2022 May 24;13(3):e01255-22. doi: 10.1128/mbio.01255-22 (PMC9239180; doi:10.1128/mbio.01255-22)
Supplement: FIG S1 [file mbio.01255-22-s0001.pdf]

A

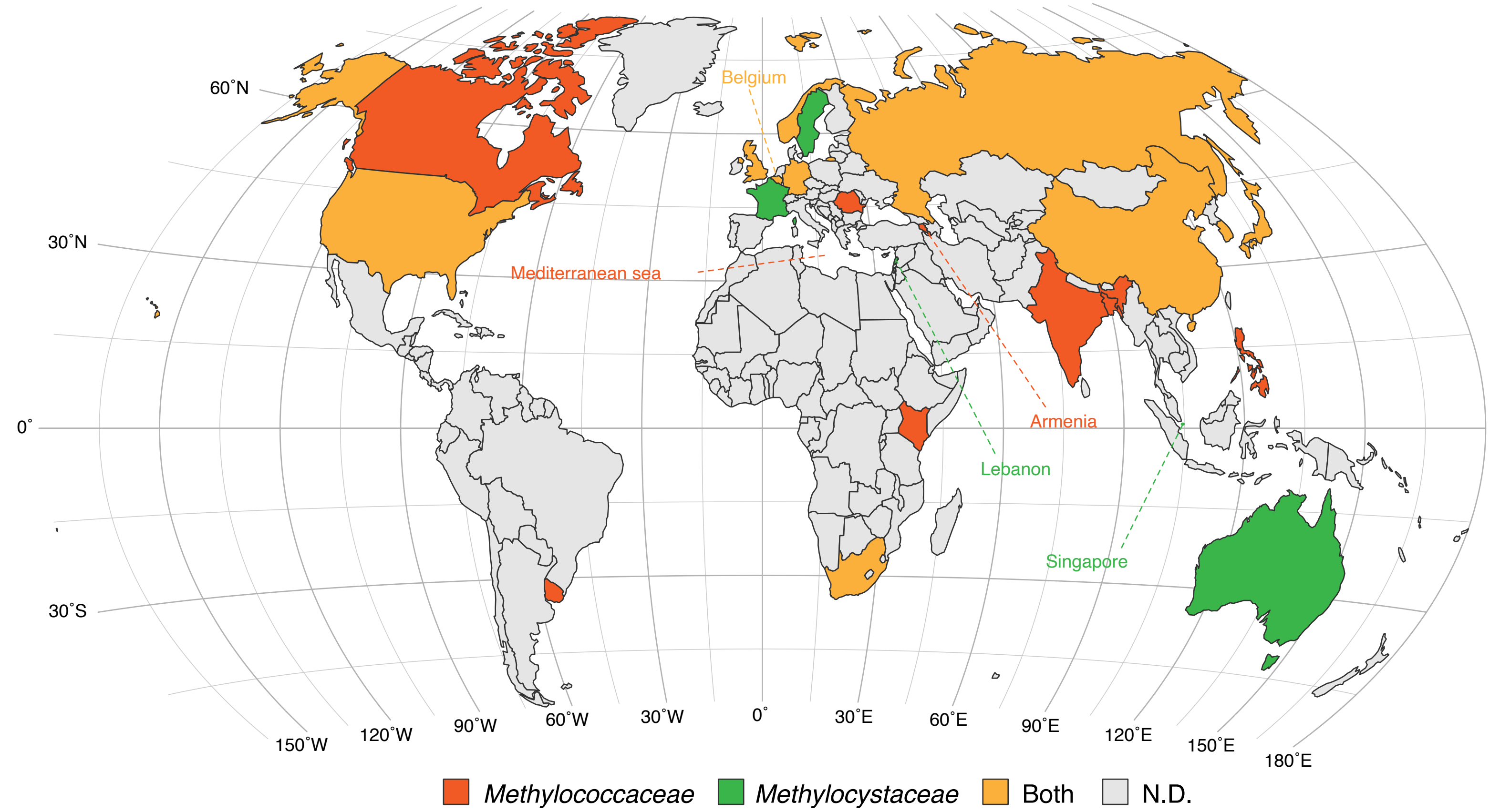

B **The isolates/detections of both *Methylococcaceae* and *Methylocystaceae* including Type I and II Methanotrophs, respectively, from different environment categorized as terrestrial, freshwater, sea, and other**

Area	Country	Family	Terrestrial	Freshwater	Sea	Other
Asia	Armenia	*Methylococcaceae*		✓		
	Bangladesh	*Methylococcaceae*	✓			
	China	*Methylococcaceae*	✓			
		Methylocystaceae	✓			
	India	*Methylococcaceae*	✓	✓		
	Japan	*Methylococcaceae*	✓		✓	
		Methylocystaceae	✓	✓		
	Lebanon	*Methylocystaceae*				✓
	Philippines	*Methylococcaceae*	✓			
	Russia	*Methylococcaceae*	✓	✓		
		Methylocystaceae	✓	✓		
	Singapore	*Methylocystaceae*	✓			
	South Korea	*Methylococcaceae*	✓	✓	✓	
		Methylocystaceae		✓		
Oceania	Australia	*Methylocystaceae*				✓
Europe	Belgium	*Methylococcaceae*		✓		✓
		Methylocystaceae		✓		
	France	*Methylocystaceae*	✓		✓	
	Germany	*Methylococcaceae*		✓		
		Methylocystaceae		✓		✓
	Norway	*Methylococcaceae*	✓	✓		
		Methylocystaceae	✓	✓		
	Romania	*Methylococcaceae*	✓			
	Sweden	*Methylocystaceae*	✓			
	United Kingdom	*Methylococcaceae*	✓		✓	✓
		Methylocystaceae	✓	✓		
North America	Canada	*Methylococcaceae*	✓	✓		
	USA	*Methylococcaceae*	✓	✓	✓	✓
		Methylocystaceae	✓	✓		✓
South America	Uruguay	*Methylococcaceae*	✓			
Africa	Kenya	*Methylococcaceae*		✓		
	South Africa	*Methylococcaceae*				✓
		Methylocystaceae				✓
Ocean	Mediterranean Sea	*Methylococcaceae*			✓	
