# Supplementary material for: In Vivo Evidence of Single 13C and 15N Isotope–Labeled Methanotrophic Nitrogen-Fixing Bacterial Cells in Rice Roots
Source: mBio. 2022 May 24;13(3):e01255-22. doi: 10.1128/mbio.01255-22 (PMC9239180; doi:10.1128/mbio.01255-22)
Supplement: TABLE S1 [file mbio.01255-22-s0007.pdf]

Table S1. Presence/absence of methane monooxygenase and nitrogen fixation structural genes (*nifHDK*) in available genomes of methanotrophs.

	NO. of genomes	probe match	methane monooxygenase		nitrogen fixation			
		Ma450	pMMO	-pMMO	nifHDK	-nifH	-nifD	-nifHDK
***Methylococcaceae* (Type I methanotrophs)**								
Methylicorpusculum	1		1		1			
Methylobacter	10		10		9		1	
Methylocaldum	8		7	1	8			
Methylococcus	10		10		10			
Methylocucumis	1		1		1			
Methylogaea	2		2		2			
Methyloglobulus	4		4		1			3
Methylomicrobium	5		5		2			3
Methylomonas	27		27		27			
Methyloprofundus	1		1		1			
Methylosarcina	1		1					1
Methyloterricola	1		1		1			
Methylotetracoccus	1		1		1			
Methylotuvimicrobium	4		4		3			1
Methylovulum	4		4		4			
**Total in *Methylococcaceae***	**80**		**79 (98.8%)**	**1 (1.3%)**	**71 (88.8%)**			
***Methylocystaceae* (Type II methanotrophs)**								
Methylocystis	22	21	22		21	1		
Methylosinus	15	15	15		15			

**Total in *Methylocystaceae***	**37**	**36 (98.3%)**	**37 (100%)**		**36 (98.3%)**	**1 (2.7%)**
