# Supplementary material for: In Vivo Evidence of Single 13C and 15N Isotope–Labeled Methanotrophic Nitrogen-Fixing Bacterial Cells in Rice Roots
Source: mBio. 2022 May 24;13(3):e01255-22. doi: 10.1128/mbio.01255-22 (PMC9239180; doi:10.1128/mbio.01255-22)
Supplement: TEXT S1 [file mbio.01255-22-s0006.pdf]

Supplementary Materials and Methods for

"*In vivo* evidence of single ^{13}C and ^{15}N isotope–labeled methanotrophic nitrogen-fixing bacterial cells in rice roots"

Shintaro Hara*, Naohisa Wada*, Sliver Sung-Yun Hsiao†, Meng Zhang, Zhihua Bao, Yoshiyuki Iizuka, Der-Chuen Lee, Shusei Sato, Sen-Lin Tang†, Kiwamu Minamisawa†

* Co-first authors: Shintaro Hara and Naohisa Wada contributed equally to this work. Author order was determined by drawing straws.

† Co-corresponding authors: syhsiao@gate.sinica.edu.tw, sltang@gate.sinica.edu.tw, and kiwamu.minamisawa.e6@tohoku.ac.jp

Genome analysis of Methanotroph

The genome sequences of 83 Methylococcaceae isolates and 44 Methylocystaceae isolates were downloaded from NCBI RefSeq (last accessed March, 2022), then coding sequences were detected by Dfast (1). The genes of pmmo, smmo, and nifHDK were retrieved by blastp search with an E-value threshold of 10^{-10}. *Methylosinus trichosporium* OB3b or *Methylococcus capsulatus* str. Bath were used as references.

Investigation of geographical and habitat distribution of methanotrophs based on source of the nitrogenase reductase *nifH* gene

We retrieved a total of 358 *nifH* gene sequences (*Methylococcaceae* [type I methanotrophs]: 176 sequences and *Methylocyctaceae* [type II methanotrophs]: 182 sequences) from nr-database of NCBI (National Center for Biotechnology Information) (last accessed Mar. 2020), and manually obtained the meta-information of sequence source including country, host association, and environment on the sequences from the NCBI and literature reviews.

Rice cultivation in a paddy field

Rice (*Oryza sativa* L. cv. Nipponbare) seedlings were prepared as described previously (2) and then transplanted on 18 June 2019 in a paddy field at the Kashimadai Experimental Station, Tohoku University (38°27′′37′N, 141°5′′33′E) under low N fertilization (2). The field was fertilized with phosphorus (P_2O_5) and potassium (K_2O) at a rate of 30 kg ha^{-1} each without N application from 2004 onward (2). Rice plants were cultivated under waterlogged conditions (water depth, 30 cm).

Stable isotope incorporation into root tissue from $^{13}CH_4$ and $^{15}N_2$ in the presence and absence of methane oxidization inhibitor

Four rice plants were harvested randomly on 26 August 2019. The entire roots were sampled from the paddy field and washed with tap water. Approximately 30 g (fresh weight) of the subsamples was placed in plastic tubes, which were introduced into two

gas-tight chambers (9.3 L each). The gas phase was replaced three times with Ar gas in a vacuum line, and then $^{15}N_2$ (40.8 atom%; SI Science, Tokyo, Japan), $^{13}CH_4$ (99 atom%; SI Science), O_2, and Ar were introduced into both chambers to achieve (v/v) 39% $^{15}N_2$, 5% CH_4, and 5% O_2 in Ar balance. Difluoromethane (CF_2H_2, DFM; Takachiho, Tsukuba, Japan), an inhibitor of methane monooxygenase, was added into one chamber at a final concentration of 0.5% (v/v). After static incubation at 25 °C for 24 h in the dark, the root samples were dried at 70 °C for 3 days and then powdered in Multi-Beads Shocker (Yasui Kikai, Osaka, Japan). The negative control of roots (0 h) was sampled just before the incubation. $^{13}C/^{15}N$ atom% concentrations and total C/N contents of the powdered root tissues were determined by using an elemental analyzer/isotope ratio mass spectrometer (Flash EA1112-Delta V Advantage ConFlo IV System; Thermo Fisher Scientific, Waltham, MA, USA).

Preparation of bacterial cells for FISH/NanoSIMS analyses

Four rice plants were harvested on 30 August 2019, and washed roots were obtained as described above. Approximately 30 g (fresh weight) of subsamples were placed into a plastic bag assembly (approximately 1 L) with a gas inlet port. The bags were closed with a heat sealer, and the gas phase was replaced three times with He gas in a vacuum line, and then $^{15}N_2$ (99.4 atom%; SI Science), $^{13}CH_4$, O_2, and He were introduced into the closed plastic bag to achieve (v/v) 33% $^{15}N_2$, 6% $^{13}CH_4$, and 12% O_2 in He balance. After static incubation at 25 °C for 23 and 42 h in the dark, the root samples were frozen in liquid nitrogen and stored at −80 °C until use. The negative control of roots (0 h) was

sampled just before the incubation. ^{13}C and ^{15}N concentrations in the root samples were determined as described above.

Bacterial cell enrichment for FISH/NanoSIMS analysis

The bacterial cells were prepared from the rice roots by the bacterial cell enrichment (BCE) method with some modification (3). Ten grams of the frozen root tissue and a metal cone (MC-100R(S), Yasui Kikai, Osaka, Japan) in a 100-mL tube (ST-10010PCR(S), Yasui Kikai) were pretreated in liquid nitrogen and ground at 2500 rpm for 10 s using Multi-Beads Shocker (MB1200, Yasui Kikai). The metal cone was removed, and then 40 mL of BCE buffer (50 mM Tris–HCl [pH 7.5], 1% Triton X-100, 2 mM 2-mercaptoethanol) was added. The powdered plant tissue in BCE buffer was homogenized with chrome steel beads (φ 6.35 mm) at 1500 rpm for 3 min using Multi-Beads Shocker. The homogenate was filtered through a layer of sterilized Miracloth (CalBiochem, La Jolla, CA, USA). The filtrate was then transferred to a 50-mL tube and centrifuged at 500 ×*g* (1800 rpm) for 5 min at 10 °C. The supernatant was transferred to a new 50-mL tube without disturbing the loose pellet and centrifuged at 8000 ×*g* (6000 rpm) for 10 min at 10 °C. The supernatant was discarded, and the pellet was suspended in 20 mL of BCE buffer and centrifuged at 8000 ×*g* (10,000 rpm) for 10 min at 10 °C. The steps of high-speed centrifugation and suspension were repeated until the supernatant became clear. The final pellet was suspended in 3 mL of phosphate-buffered saline (PBS) (g/L: NaCl, 8.0; KCl, 0.2; Na_2PO_4, 1.44; NaH_2PO_4, 0.2; pH 7.0). The suspension was overlaid on 2 mL of Nycodenz (AXIS-SHIELD PoC AS, Oslo, Norway)

solution (8 g of Nycodenz dissolved in 10 mL of PBS) and centrifuged at 8900 rpm using a swinging-bucket rotor (SE 41 Ti, Beckman Coulter, Brea, CA, USA) for 40 min at 10 °C. After centrifugation, the whitish band located at the interface of the upper and lower phases was collected as a bacterial cell fraction. The bacterial suspension was mixed with an equal volume of sterilized water, then centrifuged at 10,000 rpm for 1 min at 10 °C.

Amplicon sequencing of the 16S rRNA gene of the extracted bacteria

The bacterial pellet was used for cell observation and DNA extraction. For DNA extraction, the bacterial pellet from 0-h root samples was treated with DNase I (Takara, Japan) for 20 min at 37 °C to degrade the remaining plant DNA (4), and total DNA was prepared using a Fast Spin Kit for Soil (MP Biomedicals, Solon, OH, USA) according to the manufacturer's instructions. The bacterial community profiling was performed by amplicon sequencing of partial 16S rRNA gene, as previously described (5). In brief, the V3-4 region of 16S rRNA genes was amplified and sequenced using the MiSeq platform (Illumina, Hayward, CA, USA). Obtained sequence data were analyzed using the QIIME2 pipeline, version 2020.2 (6), and dada2 (7). The constructed feature table of an amplicon sequence variant (ASV) was assigned to the RDP database version 11.5 (8). After filtering of contaminated plant reads, 37,468–47,637 sequences remained per sample and were rarefied to 20,000 reads per sample. All raw sequence data are available in the DDBJ Sequence Read Archive (DRR325205-DRR325207).

The 16S rRNA gene of each genome were aligned with ASV of Methanotrophs by MUSCLE algorithm (9) with default parameters using MEGA11 software (10). Then phylogenetic trees were constructed with the neighbor-joining method by MEGA11 software. To evaluate tree topologies, bootstrap values were calculated with 1,000 replicates. Phylogenies were visualized with iTOL v5 (11).

Fluorescence *in situ* hybridization.

The extracted bacterial cells were fixed with 4% paraformaldehyde in 1× PBS at 4 °C overnight. The bacterial suspension was placed on indium tin oxide (ITO)-coated glass that secondly was coated with poly-L-lysine. After permeabilization with lysozyme (10 mg/mL), fluorescence *in situ* hybridization (FISH) on enriched bacterial cells was conducted as described by Wada et al. (12) using rRNA probes EUB338mix (13) and Ma450 (14) labeled with Cy3 and Alexea488, respectively. The two probes were tested for specificity using the web-based tool "RDP Probe Match" (http://rdp.cme.msu.edu/probematch/search.jsp) (last accessed Feb. 2022). As a result, the *in-silico* coverage of both EUB338mix and Ma450 probes were estimated to be complementary to 92.84% of domain Bacteria and 85.28% of the assigned *Methylocystaceae* (type II methanotrophs including *Methylocystis* 91.28%, *Methylosinus* 90.00%, and unclassified *Methylocystaceae* 77.95%), respectively. All samples were mounted by antifade mounting solution (SlowFadeTM Diamond Antifade Mountant, Thermo Fisher Scientific). The images were acquired with confocal microscopy (LSM710, Zeiss, Germany). After the acquisition, the enriched bacterial cells on ITO glasses were washed with 80% ethanol

and air-dried overnight. A negative control was performed using the complement to the EUB probes (Non338) (15).

NanoSIMS analysis

The isotopic composition of bacterial cells was analyzed using a NanoSIMS 50 L (Cameca-Ametek, Gennevilliers, France) housed in Academia Sinica, Taiwan. Secondary ions of $^{12}C^-$, $^{13}C^-$, $^{12}C^{14}N^-$, $^{12}C^{15}N^-$, and $^{32}S^-$ were collected simultaneously by multiple electron multipliers. Each 40-µm square area on ITO glass was pre-sputtered by a 150 pA Cs^+ ion beam for 1 min and sputtered by 3.2–3.7 pA Cs^+ ion beam for 30–60 min to obtain image data at 512 × 512 pixels resolution. A 30-µm and 150-µm entrance slit and aperture slit were respectively used to reach a mass resolving power of 5000 on $^{12}C^{14}N^-$ peak. The images and isotopic ratios of the cells were processed by L'Image software (developed by Larry Nittler, Carnegie Institution of Washington, Washington D.C., USA). Regions of type II methanotroph cells and other microbial cells were circled manually based on FISH images (Fig. 2A). For each time point, more than 60 cells were analyzed (type II methanotrophs and other eubacteria, n = 32 cells and n = 32 cells at 0 h, n = 35 cells and n = 34 cells at 23 h, and n = 39 cells and n = 34 cells at 42 h, respectively). The ^{13}C atom% and ^{15}N atom% ratio of each cell was calculated using the following equations:

$$^{15}N \text{ atom\%} = {}^{12}C^{15}N / ({}^{12}C^{14}N + {}^{12}C^{15}N)$$

$$^{13}C \text{ atom\%} = {}^{13}C / ({}^{13}C + {}^{12}C),$$

in which $^{12}C^{14}N$, $^{12}C^{15}N$, ^{12}C, and ^{13}C are total counts of secondary ions $^{12}C^{14}N^-$,

$^{12}C^{15}N^-$, $^{12}C^-$, and $^{13}C^-$, respectively.

After NanoSIMS analysis, the secondary electron images of bacterial cells were observed by using a field emission scanning electron microscope (JSM-7100F, JEOL, Tokyo, Japan), with 5 kV acceleration voltage and 100 pA electron beam current, for representative FISH-NanoSIMS images at each time point. To check the difference of isotopic composition between type II methanotrophs and other microorganisms, Student's *t*-test was calculated between type II methanotrophic bacteria and other eubacteria (Excel, Microsoft, Redmond WA, USA). To analyze how CH_4 assimilation corresponded to N_2 fixation at the single-cell level, linear regression analysis was performed with statistical software R v.3.6.1 (16).

SI References

1. Tanizawa Y, Fujisawa T, Nakamura Y. 2018. DFAST: a flexible prokaryotic genome annotation pipeline for faster genome publication. Bioinformatics 34:1037–1039.

2. Ikeda S, Sasaki K, Okubo T, Yamashita A, Terasawa K, Bao Z, Liu D, Watanabe T, Murase J, Asakawa S, Eda S, Mitsui H, Sato T, Minamisawa K. 2014. Low nitrogen fertilization adapts rice root microbiome to low nutrient environment by changing biogeochemical functions. Microbes Environ 29:50–59.

3. Ikeda S, Kaneko T, Okubo T, Rallos LEE, Eda S, Mitsui H, Sato S, Nakamura Y, Tabata S, Minamisawa K. 2009. Development of a bacterial cell enrichment method and its application to the community analysis in soybean stems. Microb Ecol 58:703–714.

4. Hara S, Morikawa T, Wasai S, Kasahara Y, Koshiba T, Yamazaki K, Fujiwara T, Tokunaga T, Minamisawa K. 2019. Identification of Nitrogen-Fixing Bradyrhizobium Associated With Roots of Field-Grown Sorghum by Metagenome and Proteome Analyses. Front Microbiol 10:407.

5. Hara S, Matsuda M, Minamisawa K. 2019. Growth Stage-dependent Bacterial Communities in Soybean Plant Tissues: Methylorubrum Transiently Dominated in the Flowering Stage of the Soybean Shoot. Microbes Environ 34:446–450.

6. Caporaso JG, Kuczynski J, Stombaugh J, Bittinger K, Bushman FD, Costello EK, Fierer N, Peña AG, Goodrich JK, Gordon JI, Huttley GA, Kelley ST, Knights D, Koenig JE, Ley RE, Lozupone CA, McDonald D, Muegge BD, Pirrung M, Reeder J, Sevinsky JR, Turnbaugh PJ, Walters WA, Widmann J, Yatsunenko T, Zaneveld J, Knight R. 2010. QIIME allows analysis of high-throughput community sequencing data. Nat Methods 7:335–336.

7. Callahan BJ, McMurdie PJ, Holmes SP. 2017. Exact sequence variants should replace operational taxonomic units in marker-gene data analysis. ISME J 11:2639–2643.

8. Cole JR, Wang Q, Fish JA, Chai B, McGarrell DM, Sun Y, Brown CT, Porras-Alfaro A, Kuske CR, Tiedje JM. 2014. Ribosomal Database Project: data and tools for high throughput rRNA analysis. Nucleic Acids Res 42:D633–D642.

9. Edgar RC. 2004. MUSCLE: multiple sequence alignment with high accuracy and high throughput. Nucleic Acids Res 32:1792–1797.

10. Tamura K, Stecher G, Kumar S. 2021. MEGA11: Molecular Evolutionary Genetics Analysis Version 11. Mol Biol Evol 38:3022–3027.

11. Letunic I, Bork P. 2021. Interactive Tree Of Life (iTOL) v5: an online tool for phylogenetic tree display and annotation. Nucleic Acids Res 49:W293–W296.

12. Wada N, Pollock FJ, Willis BL, Ainsworth T, Mano N, Bourne DG. 2016. In situ visualization of bacterial populations in coral tissues: pitfalls and solutions. PeerJ 4:e2424.

13. Daims H, Brühl A, Amann R, Schleifer KH, Wagner M. 1999. The domain-specific probe EUB338 is insufficient for the detection of all Bacteria: development and evaluation of a more comprehensive probe set. Syst Appl Microbiol 22:434–444.

14. Eller G, Stubner S, Frenzel P. 2001. Group-specific 16S rRNA targeted probes for the detection of type I and type II methanotrophs by fluorescence in situ hybridisation. FEMS Microbiol Lett 198:91–97.

15. Wallner G, Amann R, Beisker W. 1993. Optimizing fluorescent in situ hybridization with rRNA-targeted oligonucleotide probes for flow cytometric identification of microorganisms. Cytometry 14:136–143.

16. R Core Team. 2020. R: A Language and Environment for Statistical Computing. Vienna, Austria: R Foundations for Statistical Computing. https://www.R-project.org/.
