# Supplementary material for: In Vivo Evidence of Single 13C and 15N Isotope–Labeled Methanotrophic Nitrogen-Fixing Bacterial Cells in Rice Roots
Source: mBio. 2022 May 24;13(3):e01255-22. doi: 10.1128/mbio.01255-22 (PMC9239180; doi:10.1128/mbio.01255-22)
Supplement: TABLE S2 [file mbio.01255-22-s0008.pdf]

Table S2. Nitrogen fixation in rice roots estimated by ^{15}N enrichment and N content.[a]

Gas phase	^{15}N concentration				N_2 fixation rate [b]	
	atom%		atom% excess		μmol-N_2 g^{-1} day^{-1}	
None	0.367 ± 0.00	C				
$^{13}CH_4$ + $^{15}N_2$ + DFM	0.398 ± 0.00	B	0.032 ± 0.005	B	0.26 ± 0.03	B
$^{13}CH_4$ + $^{15}N_2$	0.457 ± 0.01	A	0.090 ± 0.010	A	0.75 ± 0.09	A
Estimated methanotrophic N_2 fixation					0.49	

DFM, difluoromethane (CF_2H_2)

[a] Data are mean ± SEM (n = 4). N_2 fixation was based on the difference of ^{15}N concentrations between root systems with and without isotope gas phase (None). Means with the same letters were not significantly different according to Turkey's HSD test ($p < 0.05$).

[b] N_2 fixation rate (b) was calculated respectively for four datasets of each replicate according to the following formula: b (μmol-N_2 [g dry weight]$^{-1}$ day^{-1}) = ($c \times 10^{-2}$) × 10^6 × ^{15}N concentration (atom% excess) / (40.8 – 0.367) (atom%) × 1/28.8, where root N content (c) was 0.95 ± 0.03 (N% of total dry weight).
