# Supplementary figures and images for: In Vivo Evidence of Single 13C and 15N Isotope–Labeled Methanotrophic Nitrogen-Fixing Bacterial Cells in Rice Roots
Source: mBio. 2022 May 24;13(3):e01255-22. doi: 10.1128/mbio.01255-22 (PMC9239180; doi:10.1128/mbio.01255-22)

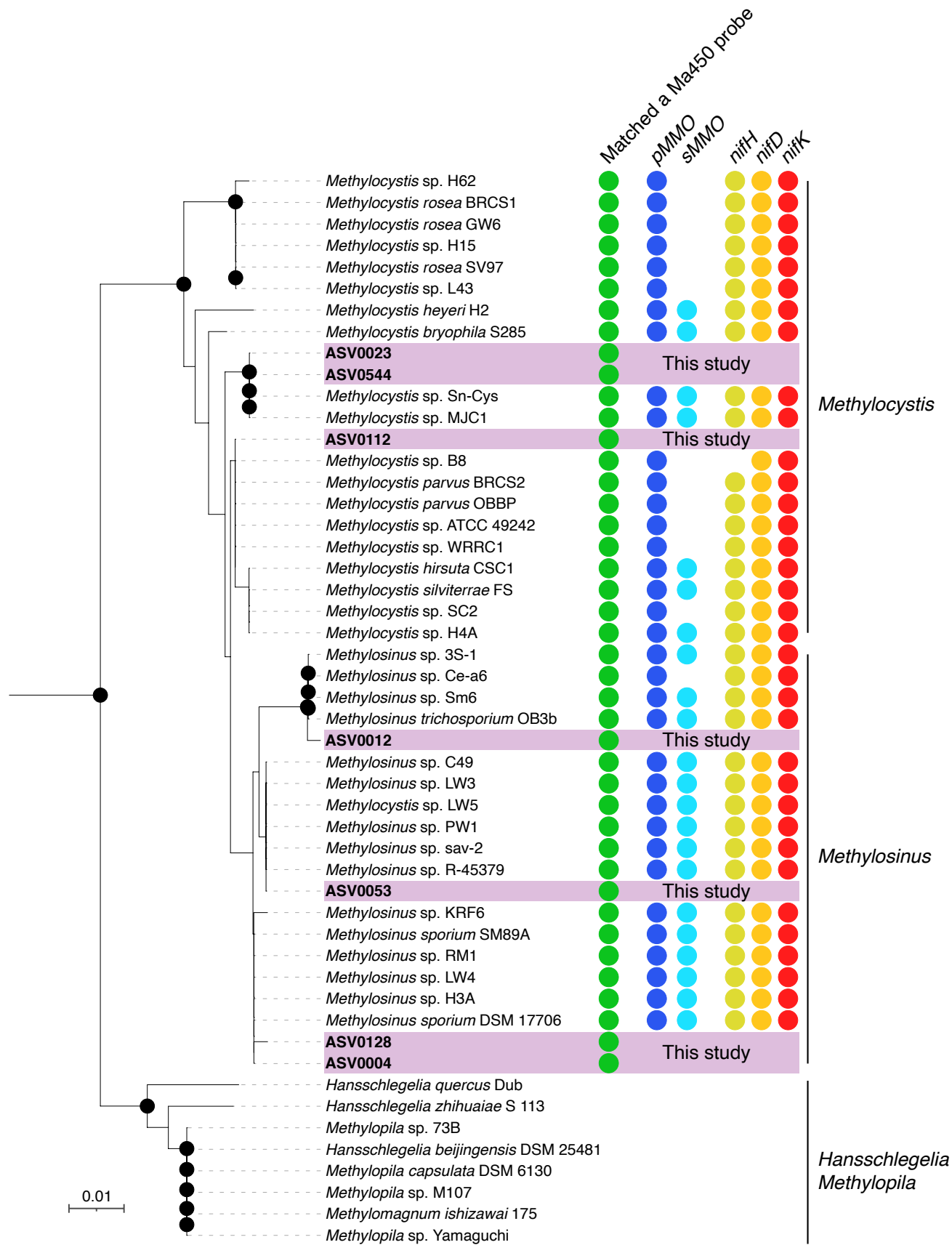

Supplement: FIG S2 [file mbio.01255-22-s0002.pdf]

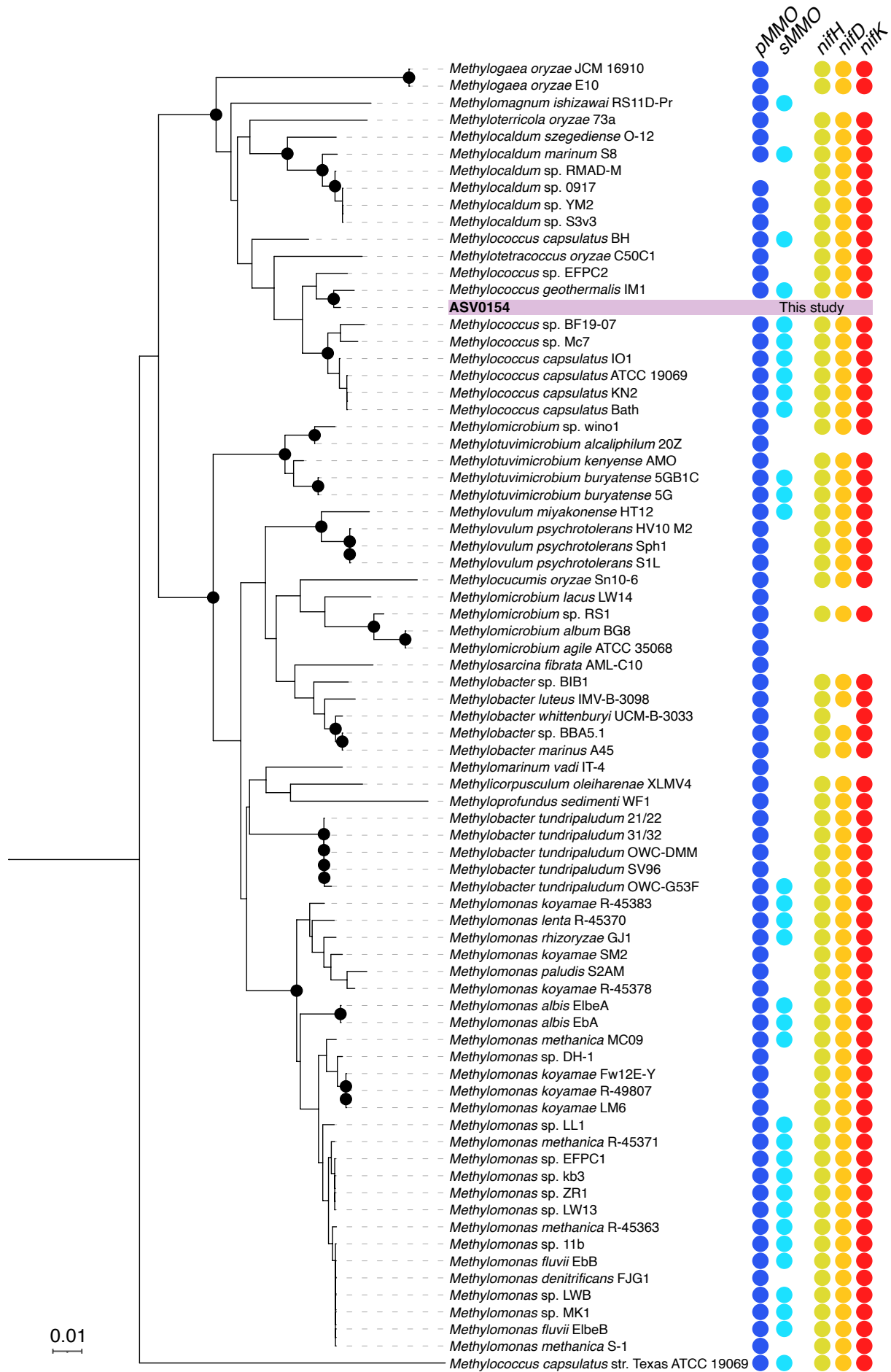

Supplement: FIG S3 [file mbio.01255-22-s0003.pdf]

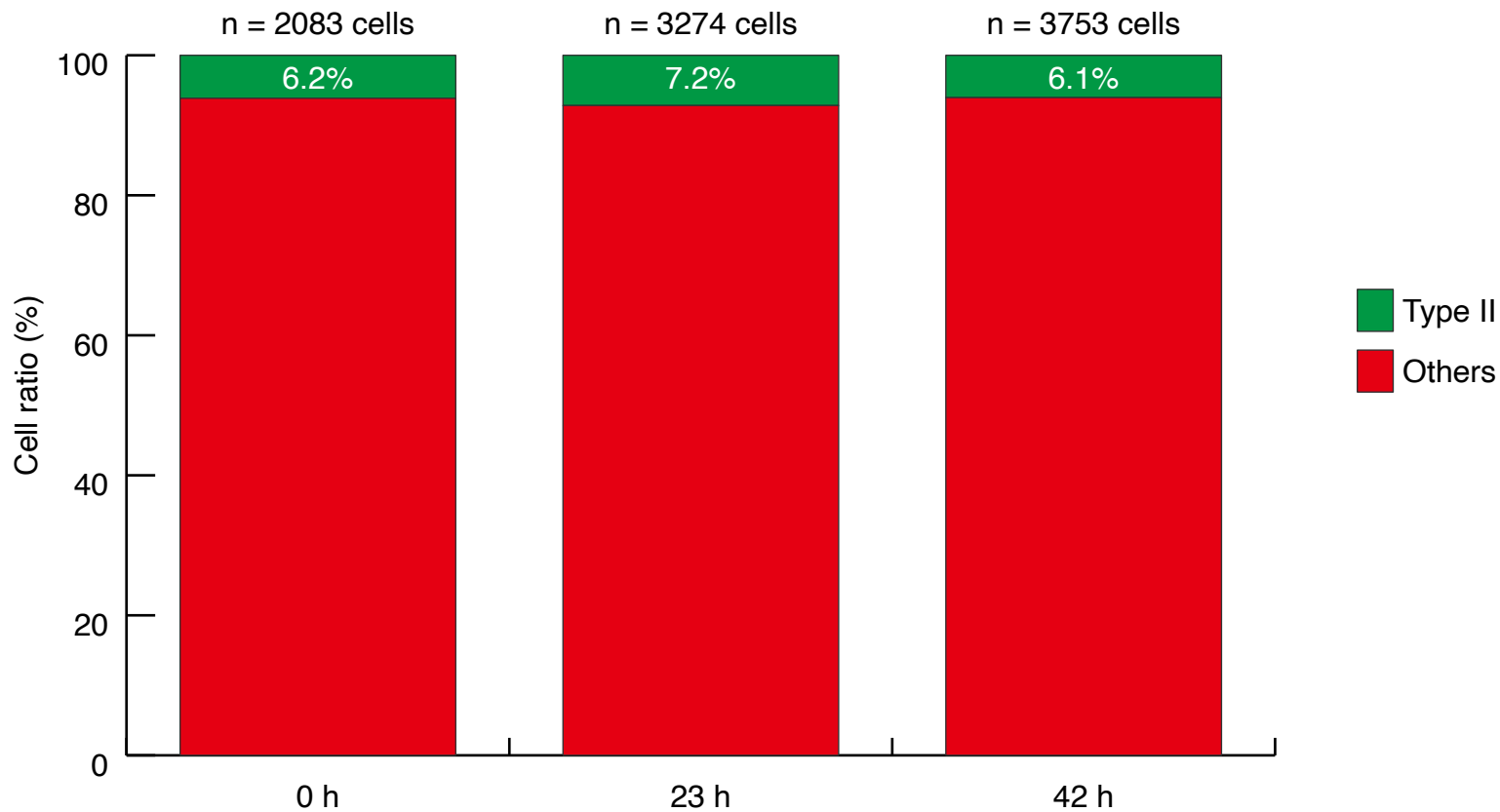

Supplement: FIG S4 [file mbio.01255-22-s0004.pdf]

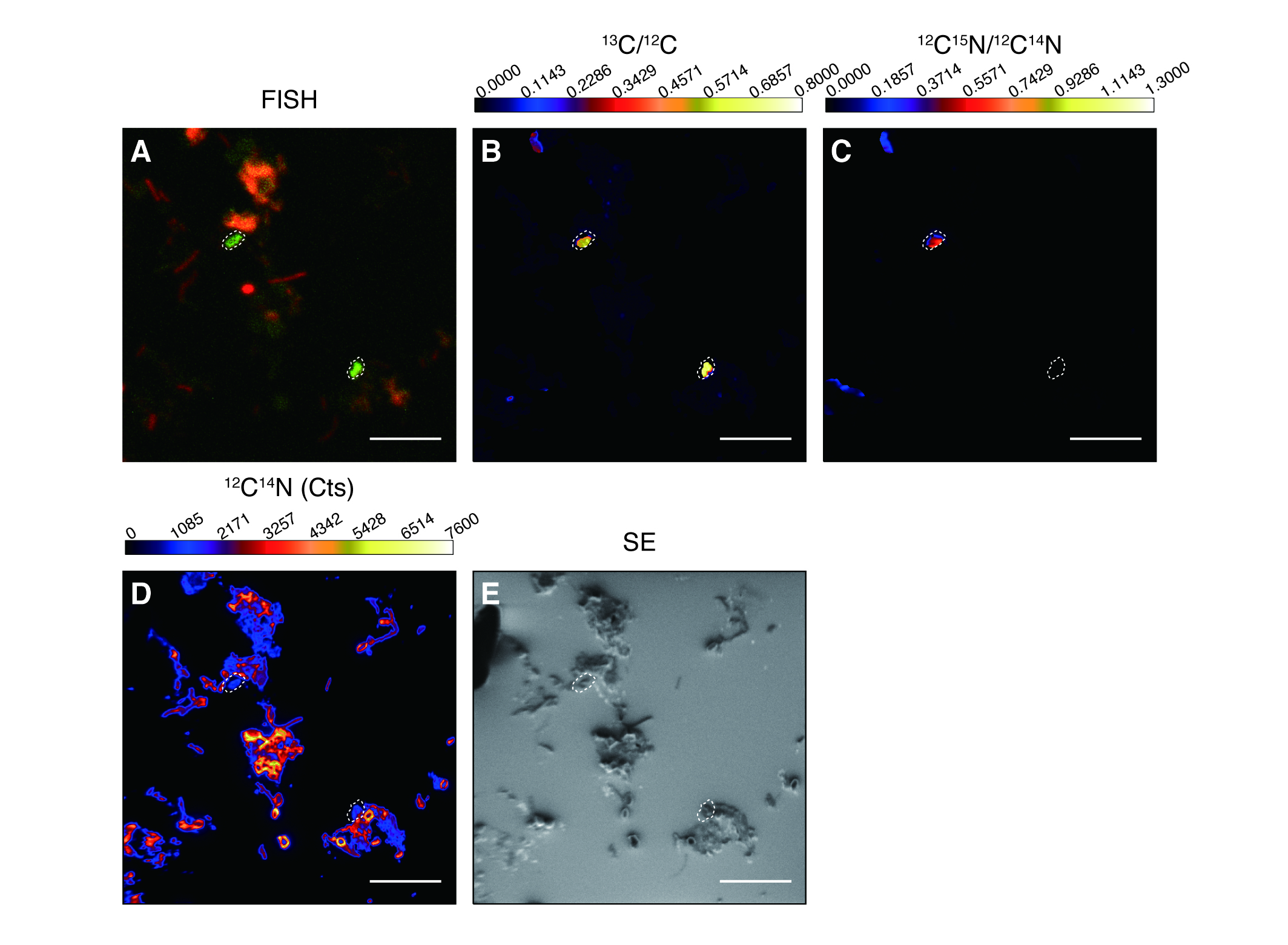

Supplement: FIG S5 [file mbio.01255-22-s0005.jpg]
